# The Interleukin-10 Family of Cytokines and Their Role in the CNS

**DOI:** 10.3389/fncel.2018.00458

**Published:** 2018-11-27

**Authors:** Amanda R. Burmeister, Ian Marriott

**Affiliations:** Department of Biological Sciences, The University of North Carolina at Charlotte, Charlotte, NC, United States

**Keywords:** interleukin-10, IL-19, IL-20, IL-22, IL-24, microglia, astrocytes, neuroinflammation

## Abstract

Resident cells of the central nervous system (CNS) play an important role in detecting insults and initiating protective or sometimes detrimental host immunity. At peripheral sites, immune responses follow a biphasic course with the rapid, but transient, production of inflammatory mediators giving way to the delayed release of factors that promote resolution and repair. Within the CNS, it is well known that glial cells contribute to the onset and progression of neuroinflammation, but it is only now becoming apparent that microglia and astrocytes also play an important role in producing and responding to immunosuppressive factors that serve to limit the detrimental effects of such responses. Interleukin-10 (IL-10) is generally considered to be the quintessential immunosuppressive cytokine, and its ability to resolve inflammation and promote wound repair at peripheral sites is well documented. In the present review article, we discuss the evidence for the production of IL-10 by glia, and describe the ability of CNS cells, including microglia and astrocytes, to respond to this suppressive factor. Furthermore, we review the literature for the expression of other members of the IL-10 cytokine family, IL-19, IL-20, IL-22 and IL-24, within the brain, and discuss the evidence of a role for these poorly understood cytokines in the regulation of infectious and sterile neuroinflammation. In concert, the available data indicate that glia can produce IL-10 and the related cytokines IL-19 and IL-24 in a delayed manner, and these cytokines can limit glial inflammatory responses and/or provide protection against CNS insult. However, the roles of other IL-10 family members within the CNS remain unclear, with IL-20 appearing to act as a pro-inflammatory factor, while IL-22 may play a protective role in some instances and a detrimental role in others, perhaps reflecting the pleiotropic nature of this cytokine family. What is clear is that our current understanding of the role of IL-10 and related cytokines within the CNS is limited at best, and further research is required to define the actions of this understudied family in inflammatory brain disorders.

## Introduction

Inflammation within the central nervous system (CNS) has devastating consequences. While it was once thought that the brain is a victim organ of infiltrating leukocytes, it is now appreciated that resident brain cells play a critical role in the initiation and/or progression of inflammatory responses within the CNS that contribute to disease states. Resident CNS cells, such as microglia and astrocytes, are able to recognize and respond to either pathogen associated molecular patterns (PAMPs) or damage associated molecular patterns (DAMPs) via their expression of innate immune pattern recognition receptors (PRRs; Bowman et al., 2003; Tsung et al., 2014; Crill et al., 2015; Serramía et al., 2015). Similar to other myeloid immune cells such as macrophages, microglia express an array of cell surface, endosomal and cytoplasmic PRRs, allowing them to rapidly respond to the presence of PAMPs and DAMPs in the extracellular milieu and within the cytosol. In addition to the well-studied Toll-like and nucleotide-binding oligomerization domain (NOD)-like families of receptors (TLR and NLR, respectively), more recent work has demonstrated the ability of these sentinel cells to functionally express molecules that serve as cytosolic sensors for foreign and/or damaged nucleic acid motifs that include DNA-dependent activator of interferon-regulatory factors (DAI), retinoic acid-inducible gene (RIG)-like receptors (RLR) and cyclic guanosine monophosphate-adenosine monophosphate synthase (cGAS; Bowman et al., 2003; Liu et al., 2010; Furr et al., 2011; Crill et al., 2015; Jeffries and Marriott, 2017). Interestingly, non-leukocytic CNS cells, including astrocytes, can also express such innate immune sensing molecules although, in contrast to microglia, such cells appear to constitutively express fewer PRR types and expression levels (Bsibsi et al., 2002, 2006). However, following activation or infection, astrocytes show rapid elevations in the repertoire and levels of expression of PRRs, suggesting that these cells may become sensitized to the presence of danger signals (Bsibsi et al., 2002; Bowman et al., 2003; McKimmie and Fazakerley, 2005).

Acute inflammatory responses play an important role in pathogen clearance in peripheral tissues and organs, as discussed elsewhere (Ma et al., 2015; Gyurkovska and Ivanovska, 2016; Newton et al., 2016). While inflammation can similarly be protective within the CNS, such responses can have severe detrimental consequences if they are too extreme or sustained. Following activation, glial cells are capable of the rapid production of chemokines and cytokines that can alter the integrity of the blood brain barrier (BBB), recruit and activate circulating leukocytes to the site of the insult, and cause cerebral edema that increases cranial pressure which, in severe cases, can result in death due to herniation, blood clots, and subsequent ischemic stroke (Bowman et al., 2003; Liu et al., 2010; Furr et al., 2011; Barichello et al., 2012; Fayeye et al., 2013; Minkiewicz et al., 2013; Pelegrín et al., 2014; Tibussek et al., 2015; Papandreou et al., 2016; Sun et al., 2016; Shah, 2018). Microglia and astrocytes respond to PAMPs (Furr et al., 2010; Liu et al., 2010; Serramía et al., 2015; Sun et al., 2016) and DAMPs (Minkiewicz et al., 2013; Tsung et al., 2014) by releasing the signature inflammatory cytokines, interleukin-6 (IL-6) and tumor necrosis factor-α (TNF-α), and the chemokine IL-8. While these mediators can assist in the recruitment of leukocytes that include those responsible for protective adaptive immune responses, long-term exposure to these cytokines results in local tissue damage. As such, it is essential that this acute inflammatory phase is regulated and limited to prevent neurological damage. In this review article, we will discuss the ability of glial cells to produce mediators that can limit or resolve sterile or pathogen-induced neuroinflammation, with a particular emphasis on the IL-10 family of cytokines.

## CNS Cells Can Contribute to the Resolution Phase of Immune Responses Within the Brain

Inflammation is typically biphasic and features the rapid production of pro-inflammatory mediators, followed by a decrease in their release and the subsequent delayed expression of immunosuppressive factors that limit their production and/or effect (Mino and Takeuchi, 2013; Shen et al., 2013; Headland and Norling, 2015). Such a change in the cytokine expression profile during this resolution phase serves to prevent prolonged exposure to inflammatory mediators and limits associated tissue damage. The transient nature of pro-inflammatory cytokine and chemokine production by glia and leukocytes (Conti et al., 2004; Barichello et al., 2011) results, at least in part, by a modification in cytokine mRNA stability by RNA binding proteins, which bind to the adenylate-uridylate (AU)-rich elements (ARE) in the 3′ untranslated region (UTR) of the mRNA. For example, the RNA binding protein tristetraprolin (TTP) has been demonstrated to have an anti-inflammatory role as it binds to the UTR of mRNA encoding the key pro-inflammatory cytokine TNF-α, thereby destabilizing it (Liu et al., 2013; Patial et al., 2016; Astakhova et al., 2018).

In addition to factors that can limit the production of inflammatory mediators in the continued presence of activating stimuli, other components can be upregulated in glial cells or their neighbors that attenuate their effects. Anti-inflammatory response (AIR) gene products include suppressor of cytokine signaling (SOCS) molecules, and these proteins are potent inhibitors of inflammatory mediator signaling cascades (Croker et al., 2003; Hutchins et al., 2012). For example, SOCS3 functions by binding to the IL-6 family receptor subunit, gp130, and inhibiting the signal cascade for this cytokine family (Babon et al., 2014; Wilbers et al., 2017). Importantly, cytokines that are recognized to have immunosuppressive effects, including IL-4 and IL-13, can induce the expression of SOCS molecules in both peripheral immune cells and non-leukocytic cell types, thereby contributing to their anti-inflammatory effects (Hebenstreit et al., 2003; Jackson et al., 2004; Albanesi et al., 2007; Dickensheets et al., 2007). Within the CNS, activated glial cells have been shown to express members of the SOCS family of molecules and their importance has been discussed in detail in other literature reviews (Campbell, 2005; Baker et al., 2009). Additionally, soluble cytokine decoy receptors, such as decoy receptor 3 and IL-2 receptor 2 (IL-1R2), that can bind inflammatory factors and prevent their interaction with target cell receptors, can be produced during this anti-inflammatory period (Francis et al., 2001; Ichiyama et al., 2008; Liu et al., 2015; Bonecchi et al., 2016).

However, a major component in the transition of immune responses from an inflammatory to a resolution phase is the delayed secondary production of mediators that are immunosuppressive and/or neuroprotective. For example, pathogen recognition via PRRs generates a complex response that includes the production of both inflammatory mediators and factors that can restore an immunoquiescent environment, such as microRNAs (miRNAs). Once thought of as “junk” RNA that is generated during gene transcription, miRNAs have been identified to play a major role in switching off acute inflammatory responses, and several have been shown to have such functions within the CNS (Ponomarev et al., 2011; Iyer et al., 2012; Cho et al., 2015). miRNAs appear to contribute to the maintenance of an immunoquiescent environment in the CNS by reducing the production of inflammatory mediators by microglia, perivascular macrophages, and astrocytes, and by downregulating the expression of molecules involved in innate immune sensing pathways that render these cells less responsive to insult (Ponomarev et al., 2011; Iyer et al., 2012; Lai et al., 2013; Zhao et al., 2013; Cho et al., 2015; Sun et al., 2015; Qin et al., 2016).

In addition, microglia and astrocytes play a critical role in providing neurons with a protective homeostatic environment within the brain by expressing excitatory amino acid transporters (EAAT), such as glutamate transporter 1 (GLT-1; Almeida et al., 2005; Persson et al., 2005, 2007). During inflammation, extracellular glutamate levels show increases that could potentially be neurotoxic (Zou and Crews, 2005), but EAAT expression and glutamate uptake by glia are elevated, thereby protecting neurons from excitotoxicity (Moidunny et al., 2016).

Furthermore, and in contrast to the rapid production of pro-inflammatory mediators, immunosuppressive cytokines are typically produced at peripheral sites in a delayed manner to promote tissue repair. These suppressive cytokines include IL-4, IL-10, IL-13 and transforming growth factor-β (TGF-β), which can significantly reduce the level of pro-inflammatory cytokine production by activated CNS cells (Moore et al., 2001; Qian et al., 2008). In addition, these soluble mediators can alter microglial phenotype polarization from the predominantly inflammatory “M1” phenotype to a more immunoregulatory “M2” phenotype that expresses protective and/or repairing factors (Qian et al., 2008; Guglielmetti et al., 2016; Rossi et al., 2018). Of these anti-inflammatory factors, IL-10 is generally considered to be the quintessential immunosuppressive cytokine produced within the CNS.

## IL-10 Is Expressed Within the CNS and Limits Glial Inflammatory Responses

It is known that IL-10 plays a critical role in the resolution of peripheral inflammation and this molecule has been the most widely studied anti-inflammatory cytokine, as discussed in numerous reviews (Hutchins et al., 2013; Headland and Norling, 2015; Mingomataj and Bakiri, 2016). Since its initial discovery, IL-10 has been found to be produced by an array of leukocytic cell types, including monocytes and granulocytes, as well as non-immune cells such as epithelial cells and keratinocytes (Moore et al., 2001; Moser and Zhang, 2008). Importantly, isolated microglia and astrocytes produce IL-10 in a delayed manner, with increased IL-10 mRNA expression seen at 8 h after activation with TLR ligands or microbial pathogens, and detectible protein release at 24 h following stimulation (Jack et al., 2005; Bsibsi et al., 2006; Rasley et al., 2006; Park et al., 2007; Gautam et al., 2011; Werry et al., 2011; Gutierrez-Murgas et al., 2016). In addition, these resident CNS cells have been demonstrated to express IL-10 *in situ* following *in vivo* LPS challenge (Park et al., 2007).

Interestingly, such delayed IL-10 production by glia appears to occur secondary to the release of inflammatory mediators, as we have shown the rapid induction of this cytokine following exposure to conditioned media from bacterially challenged cells (Rasley et al., 2006). Furthermore, the inflammatory cytokines IL-6 and TNF-α have been demonstrated to induce IL-10 production by microglia in a dose dependent manner (Sheng et al., 1995). IL-10 production by cytokine-challenged microglia can be further augmented by neurotransmitters including glutamate (Werry et al., 2011), and damage-associated molecules such as adenosine (Koscsó et al., 2012). In contrast, the neuropeptide, substance P (SP), appears to play a role in the reduction of IL-10 levels within the CNS that occurs following bacterial infection, as this effect was not seen following prophylactic administration of an antagonist for its high affinity receptor (Chauhan et al., 2008, 2011). Such an effect suggests that SP can promote neuroinflammation in two ways, first by exacerbating pro-inflammatory glial and infiltrating leukocyte responses, as discussed in our recent review on this topic (Johnson et al., 2017), and second by limiting the expression of immunosuppressive mediator production within the brain.

**Figure 1:**
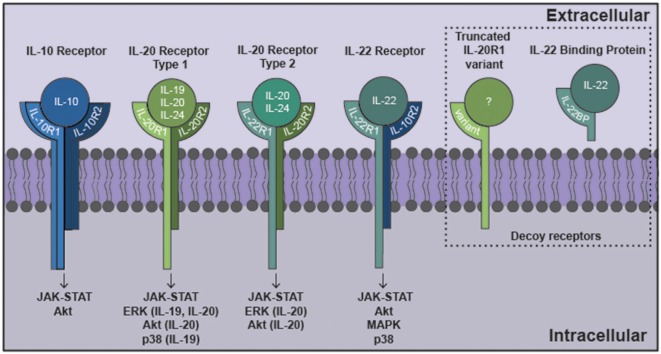
The interleukin-10 (IL-10) family of cytokines exert their effects via heterodimeric receptor subunits. IL-10 signals through a complex of two IL-10R1 and two IL-10R2 subunits. IL-22 signals via an IL-22R1 subunit in combination with an IL-10R2 subunit. IL-19 signals through the type 1 IL-20R consisting of IL-20R1 and IL-20R2 subunits. IL-20 and IL-24 can signal via either type 1 IL-20R or the type 2 IL-20R consisting of IL-22R1 and IL-20R2 subunits. Signaling through these cognate cell surface receptors initiates the activation of canonical Janus kinase (JAK)-signal transducer and activator of transcription (STAT) signaling pathways. Additionally, other signaling cascades have been identified for this family that includes ERK, Akt, mitogen activated protein kinase (MAPK) and p38. Potential decoy receptors for these cytokines include IL-22 binding protein (IL-22BP) and a truncated IL-20R1 variant that bind IL-22 and an undetermined ligand, respectively.

IL-10 exerts local effects on cells that express a receptor that are composed of two subunits, IL-10 receptor (IL-10R)1 and IL-10R2 (Moore et al., 2001) as shown in Figure 1. While most cell types are known to express IL-10R2 constitutively, IL-10R1 expression tends to be restricted to cells of hematopoietic lineage (Moore et al., 2001; Wolk et al., 2002; Moser and Zhang, 2008). As might be expected given their myeloid lineage, microglia constitutively express both IL-10R1 and IL-10R2 (Hulshof et al., 2002). More surprisingly, resting astrocytes also express both IL-10 receptor subunits (Molina-Holgado et al., 2001; Ledeboer et al., 2002; Xin et al., 2011; Perriard et al., 2015; Table 1). However, such expression by other glial cell types remains controversial with reports of IL-10R1 expression by rat oligodendrocytes (OD) but not human cells (Molina-Holgado et al., 2001; Hulshof et al., 2002), and may also be expressed by neurons (Sharma et al., 2011).

**Table 1:** Glial sources and targets of the interleukin-10 (IL-10) family within the brain.

IL-10 family member	CNS cellular source	Inducers of expression	CNS cellular target	Receptor subunits	Decoy receptor
IL-10	Microglia Astrocytes	*Neisseria meningitidis* *Borrelia burgdorferi* LPS TLR3 ligand IL-6 TNF-α Adenosine Glutamate	Microglia Astrocytes Oligodendrocytes	IL-10R1/IL-10R2 IL-10R1/IL-10R2 IL-10R1	
IL-19	Microglia Astrocytes	*Staphylococcus aureus* *Neisseria meningitidis* *Streptococcus pneumoniae* Parasitic nematode Gamma radiation LPS TLR5 ligand	Microglia Astrocytes	IL-20R2 Perhaps IL-20R1 IL-20R1-/IL-20R2	Truncated IL-20R1 variant
IL-20	Glioblastoma cells Mixed glial cells	Ischemia-hypoxia LPS	Microglia Astrocytes	IL-22R1/IL-20R2 IL-20R1/IL-20R2 IL-22R1/IL-20R2	Truncated IL-20R1 variant
IL-22	Unknown	West Nile virus TMEV IL-23 IL-6	Microglia Astrocytes BBB endothelial cells Glioblastoma	IL-22R1/IL-10R2 IL-22R1/IL-10R2 IL-22R1/IL-10R2 IL-22R1/IL-10R2	IL-22BP
IL-24	Astrocytes	Chikungunya virus *Neisseria meningitidis* LPS	Microglia Astrocytes	IL-22R1/IL-20R2 IL-20R1/IL-20R2 IL-22R1/IL-20R2	Truncated IL-20R1 variant

*LPS, Lipopolysaccharide; TLR, Toll-like receptor; TMEV, Theiler’s murine encephalomyelitis virus*.

Following IL-10 binding to its receptor, this cytokine initiates its cellular effects via a canonical Janus kinase (JAK)/signal transducer and activator of transcription (STAT) pathway that features JAK1 and STAT3, which subsequently induces the expression of genes associated with immunosuppression (Moore et al., 2001; Hutchins et al., 2013). As we have demonstrated, STAT3 is phosphorylated in murine microglia following exposure to IL-10 (Rasley et al., 2006). Similarly, STAT3 phosphorylation has been observed in cortical neurons and retinal ganglion cells (RGCs) in response to IL-10, although this cytokine has also been shown to activate other signaling components including Akt in these cells (Boyd et al., 2003; Sharma et al., 2011).

The ability of IL-10 to regulate inflammatory TH1 responses has been well characterized (Couper et al., 2008; Moser and Zhang, 2008), and it exerts its immunosuppressive effects by decreasing pro-inflammatory mediator and co-stimulatory molecule expression by leukocytes (Fiorentino et al., 1991; Ding et al., 1993). Furthermore, IL-10 has been shown to induce the expression of anti-inflammatory miRNAs that have been shown to negatively regulate signaling via TLRs and alter the stability of inflammatory cytokine mRNA (Couper et al., 2008; Moser and Zhang, 2008; Curtale et al., 2013; Wilbers et al., 2017). Consistent with this role in the periphery, IL-10 plays an important role in maintaining homeostasis within the CNS (Gutierrez-Murgas et al., 2016). It contributes to the regulation of synaptic pruning by glial cells (Lim et al., 2013; Schwartz et al., 2013; Ellwardt et al., 2016) and limits the damaging effects of neuroinflammation. Specifically, IL-10 reduces glial pro-inflammatory mediator production and reactive astrogliosis in response to the presence of pathogenic microbes or their components (Balasingam and Yong, 1996; Ledeboer et al., 2000; Rasley et al., 2006; Chang et al., 2009; Curtale et al., 2013; Hutchins et al., 2013). Furthermore, this cytokine can alter microglial and astrocyte phenotypes to those that can limit inflammation, promote the production of another immunosuppressive mediator, TGF-β by astrocytes, and induce the expression of mRNA encoding the negative regulator of cytokine signaling SOCS3 (Balasingam and Yong, 1996; Rasley et al., 2006; Norden et al., 2014).

Consistent with these *in vitro* findings, the importance of IL-10 in the regulation of neuroinflammatory damage has been demonstrated *in vivo* in animal models of CNS disorders. IL-10 deficient mice show uncontrolled inflammation and increased susceptibility to bacterial, parasitic and viral infections of the CNS (Gazzinelli et al., 1996; Gutierrez-Murgas et al., 2016; Martin and Griffin, 2017). In these studies, increased mortality was associated with elevated levels of inflammatory mediators in the absence of endogenous IL-10 expression (Gazzinelli et al., 1996). In addition to infectious CNS disorders, a role for IL-10 in limiting detrimental neuroinflammation in “sterile” autoimmune diseases, including multiple sclerosis (MS), has been suggested. Genetic polymorphisms of the IL-10 gene that result in reduced expression of this cytokine have been associated with the incidence of MS in human subjects (Martinez Doncel et al., 2002; Myhr et al., 2002; Talaat et al., 2016). Similarly, increased levels of IL-10 in the CNS have been shown to reduce disease severity in mouse models of experimental autoimmune encephalomyelitis (EAE; O’Neill et al., 2006; Klose et al., 2013).

Taken together, the available data indicates that IL-10 plays a critical role in limiting CNS inflammation in a similar manner to that seen at peripheral sites by altering the ability of resident glia and infiltrating leukocytes to respond to activating stimuli, and by reducing the production of inflammatory mediators by these cells. However, IL-10 is only one member of a family of cytokines that are grouped together based upon their similar structures and their sharing of common receptor subunits (Ouyang et al., 2011; Rutz et al., 2014). This family includes IL-19, IL-20, IL-22, and IL-24 (Ouyang et al., 2011; Rutz et al., 2014), and these IL-10 relatives are only now being recognized to exert a regulatory role within the CNS.

## IL-19 May Function in a Similar Immunosuppressive Manner to IL-10 in the CNS

Around the time that IL-10 was discovered, a number of proteins showing a high degree of homology to this cytokine were identified that were subsequently categorized as the IL-10 family. Gallagher et al. (2000) described a list of potential IL-10 family members based upon homologous gene sequences. This work led to the identification of IL-19, a IL-10 homolog expressed by bacterial LPS challenged immune cells, including monocytes and T and B lymphocytes, which can be detected at sites such as the skin following* Staphylococcus aureus* infection (Gallagher et al., 2000, 2004; Wolk et al., 2002; Reiss-Mandel et al., 2018).

Interestingly, elevated levels of mRNA encoding IL-19 have been observed in mouse brain parenchyma after gamma radiation treatment (Baluchamy et al., 2010), and three studies have shown that isolated murine astrocytes express IL-19 mRNA and protein following challenge with bacteria or their components (Cooley et al., 2014; Nikfarjam et al., 2014; Horiuchi et al., 2015; Table 1). However, the question of whether microglia are a significant source of IL-19 remains contentious, with one study reporting the ability of neonatal murine microglia to release high levels of IL-19 in response to LPS (Horiuchi et al., 2015), while our own work indicates that these cells and a murine microglial cell line express little or no mRNA encoding IL-19 following challenge with either LPS or *N. meningitidis* (Cooley et al., 2014). Importantly, IL-19 expression within the CNS and by isolated glia following challenge demonstrates delayed kinetics of induction, which is consistent with a secondary, and perhaps protective, response (Cooley et al., 2014; Nikfarjam et al., 2014). Similarly, IL-19 is produced in a delayed manner within the brain cortex of mice infected with a parasitic nematode (Yu et al., 2015), although the precise function of this cytokine in this model has not been determined.

As shown in Figure 1, IL-19 exerts its effects on cells expressing a heterodimeric receptor that consists of the subunits IL-20 receptor (IL-20R)1 and IL-20R2 (Rutz et al., 2014). While this dimeric receptor is commonly referred to as IL-20R, it has been demonstrated that IL-19 binds to the IL-20R2 subunit with a higher affinity than IL-20 (Dumoutier et al., 2001; Logsdon et al., 2012). This cognate receptor is constitutively expressed in human tissues including the pancreas, liver and skin (Wolk et al., 2002). In contrast, immune cells express the IL-20R2 subunit but fail to express IL-20R1 either at rest or following exposure to LPS (Wolk et al., 2002; Kunz et al., 2006; Ouyang et al., 2011). Consistent with this, we have demonstrated that astrocytes constitutively express both IL-20R1 and IL-20R2, while microglia exclusively possess the IL-20R2 subunit (Cooley et al., 2014; Table 1). However, it should be noted that one study has reported the contradictory finding that microglia express the cognate receptor while astrocytes express just the IL-20R2 subunit (Horiuchi et al., 2015). To date, the reason for the apparent discrepancy in these findings remains unclear. Finally, we have also detected a novel truncated IL-20R1 subunit (IL-20R1 variant) in the mouse cortical brain (Table 1) that lacks the cytoplasmic signaling tail (Cooley et al., 2014). As such, it is possible that this truncated protein serves as a decoy receptor for IL-19, and we have reported the intriguing finding that the expression of this receptor is downregulated following infection (Cooley et al., 2014), an effect that could render CNS cells more susceptible to the effects of IL-19.

Following complexing of IL-19 with IL-20R1/IL-20R2, JAK associates with the cytoplasmic tail of IL-20R1 and phosphorylates the transcription factor STAT3 (Gallagher, 2010; Wegenka, 2010; Rutz et al., 2014). Within the CNS, IL-19 has been demonstrated to activate JAK/STAT signaling in microglia, as evidenced by STAT3 phosphorylation in these cells following IL-19 exposure (Horiuchi et al., 2015). However, defining the role of IL-19 in inflammatory responses in the periphery and the CNS has been hampered by inconsistent reports. Despite these issues, and evidence that IL-19 can exert pleiotropic effects that are dependent on the target cell type and stage of the insult, the preponderance of available evidence indicates that IL-19 is immunosuppressive. Similarly, while the reported effects of IL-19 on glial immune responses have shown variability, they are generally consistent with an immunosuppressive role for this cytokine. For example, our laboratory showed that IL-19 administration increases the expression of mRNA encoding the negative regulator of inflammatory cytokine signaling, SOCS3, in primary murine astrocytes, and decreases the production of IL-6 and TNF-α by these cells following stimulation (Cooley et al., 2014). Furthermore, other investigators demonstrated that LPS challenged microglia isolated from IL-19 deficient mice produce significantly higher levels of IL-6 and TNF-α, consistent with the removal of an inhibitory effect on these cells, that was reversible with recombinant IL-19 addition (Horiuchi et al., 2015). However, it should be noted that IL-19 treatment did not exert a demonstrable effect on the production of inflammatory mediators by LPS-challenged astrocytes in this study (Horiuchi et al., 2015).

Additionally, some evidence suggests that IL-19 may serve to limit CNS damage in cases of “sterile” neuroinflammation. For example, elevations in the expression of mRNA encoding IL-19 have been noted in peripheral blood mononuclear cells in an animal model of stroke (Rodriguez-Mercado et al., 2012), and IL-19 administration prior to ischemia-reperfusion injury has been associated with decreased leukocyte activation/infiltration and lessened neurological damage (Xie et al., 2016). Finally, genetic polymorphisms in the IL-19 locus have been associated with the risk of MS development (Khodakheir et al., 2017), similar to that seen for IL-10, although mechanistic links between these and MS neuropathology have not been defined.

## IL-22 Can Exert Both Protective and Detrimental Effects Within the CNS

IL-22 was first identified as a product of cytokine-activated lymphoma cells (Dumoutier et al., 2000) and subsequent studies demonstrated that IL-22 is a major product of the TH17 subpopulation of CD4^+^ lymphocytes (Liang et al., 2006). However, IL-22 expression does not appear to be limited to T-cells as other leukocytes, including macrophages, can also express this cytokine (as reviewed in Dudakov et al., 2015). Importantly, constitutive expression of IL-22 has been described in the CNS (Zenewicz and Flavell, 2008; Dudakov et al., 2015) and immunohistochemical staining of human brain tissue has shown that IL-22 is present in both gray and white matter in healthy individuals (Perriard et al., 2015). Furthermore, IL-22 expression within the CNS has been demonstrated to increase following viral infection (Levillayer et al., 2007; Wang et al., 2012), and it is tempting to speculate that such increases result from local production of inflammatory cytokines. However, such a mechanism of induction has not been investigated, and the specific CNS cell type(s) responsible for constitutive and/or inducible IL-22 production have yet to be determined.

The functional receptor for IL-22 is a heterodimer composed of IL-22R1 and IL-10R2 (Zenewicz and Flavell, 2008; Dudakov et al., 2015; Figure 1), and binding of IL-22 to the IL-22R1 subunit allows for IL-10R2 to form a complex that initiates a signaling cascade (Dudakov et al., 2015). The IL-22 receptor is highly expressed in the pancreas, kidney, skin and liver, and expression can be further upregulated following stimuli such as *S. aureus* infection (Myles et al., 2013; Rutz et al., 2014; Dudakov et al., 2015). While early work failed to detect the presence of IL-22R1 in immune cells, subsequent studies have reported the induction of receptor subunit expression in myeloid cells following bacterial challenge and the ability of these cells to respond to IL-22 (Dhiman et al., 2009, 2014; Zeng et al., 2011). In the brain, BBB endothelial cells, astrocytes and glioblastoma cells have all been shown to constitutively express both the IL-22R1 and IL-10R2 subunits (Kebir et al., 2007; Akil et al., 2015; Perriard et al., 2015; Table 1). Interestingly, and in contrast to other myeloid cells, we have recently demonstrated that microglia constitutively express robust levels of the IL-22R1 protein (Burmeister et al., unpublished observations).

Decoy receptors are known to play an important role in regulating the effects of their associated cytokines, and IL-22 binding protein (IL-22BP) serves as a soluble decoy receptor for IL-22 by binding this cytokine with higher affinity than cell associated IL-22R1 (Martin et al., 2017). Leukocytes such as dendritic cells can release IL-22BP but the effects of higher decoy receptor expression, and hence lower levels of available IL-22, appear to vary according to the disease condition, with decreased disease severity in an animal model of psoriasis (Martin et al., 2017) and greater hepatic fibrosis in human schistosomiasis patients (Sertorio et al., 2015). These data therefore indicate that IL-22 can serve both detrimental and protective roles. In the CNS, IL-22BP expression is upregulated in the cerebral spinal fluid (CSF) of patients with active MS (Perriard et al., 2015) and mice deficient in the expression of IL-22BP show less severe disease in a mouse model of EAE (Laaksonen et al., 2014), while increased IL-22BP expression correlates with greater macrophage infiltration and more severe neuroinflammation in a rat EAE model. Together, these findings support the notion that IL-22 limits the damaging effects of CNS inflammation (Beyeen et al., 2010).

Like other IL-10 family members, binding of IL-22 to its transmembrane receptor initiates a JAK/STAT signaling cascade in target cells (Dudakov et al., 2015). Typically, tyrosine kinase 2 (Tyk2) and/or JAK1 activation is associated with IL-22 signaling, with subsequent promiscuous STAT phosphorylation (Lejeune et al., 2002). STAT1, STAT3 and STAT5 activation have all been reported following exposure to IL-22 (Lejeune et al., 2002; Dudakov et al., 2015). However, IL-22 has also been demonstrated to activate mitogen activated protein kinase (MAPK) and p38 pathways in keratinocytes and synovial fibroblasts (Ikeuchi et al., 2005; Andoh et al., 2009). Consistent with this, human glioblastoma cell lines exposed to IL-22 show increases in both STAT3 and Akt phosphorylation (Akil et al., 2015).

As reviewed elsewhere, IL-22 appears to contribute to host defense at peripheral sites (Zenewicz and Flavell, 2008; Ouyang et al., 2011; Rutz et al., 2014), while in the brain IL-22 can function as a cell survival factor as it protects glioblastoma cells from the apoptosis-inducing effects of serum starvation and Fas ligand exposure (Akil et al., 2015). Similarly, primary human astrocytes treated with IL-22 demonstrate increased survival rates following challenge with TNF-α (Perriard et al., 2015). However, IL-22 may also disrupt the integrity of BBB tight junctions by reducing the level of expression of occludin by endothelial cells, and promote the recruitment of CD4^+^ lymphocytes by elevating the production of CCL2 (MCP-1) by these BBB cells (Kebir et al., 2007). As such, IL-22 may either act in a protective manner or may exacerbate detrimental host immune responses.

Elevated IL-22 levels have been detected in the blood plasma of patients with peripheral inflammatory diseases such as psoriasis and Crohn’s disease (Wilson et al., 2010), and the severity of Guillain-Barré Syndrome (GBS) appears to correlate with CSF and plasma concentrations of this cytokine (Wilson et al., 2010). However, it is not known whether such elevations underlie these disorders or, rather, represent a compensatory response of the host to limit inflammatory damage. Within the CNS, it is similarly unclear whether IL-22 provides protection during MS/EAE (Beyeen et al., 2010; Laaksonen et al., 2014; Perriard et al., 2015). Increased levels of IL-22 protein have been reported in the serum, but not the CSF, of patients with active MS (Perriard et al., 2015), while IL-22 has been found to be expressed in the CNS early in the development of EAE in the rat (Almolda et al., 2011). The finding that IL-22 expression diminishes during resolution in this rodent model has been taken as an indication that it contributes to the inflammatory phase of this MS-like disease (Almolda et al., 2011). However, it is important to note that mice lacking IL-22 show no significant difference in the level of EAE-associated neuroinflammation, suggesting that this cytokine is not a major driving force for disease development (Kreymborg et al., 2007).

In an animal model of West Nile virus associated encephalitis, mice lacking IL-22 fail to show significant differences in protective IFN-β expression, but do exhibit elevated levels of the key inflammatory cytokines, TNF-α and IL-6, and have higher viral loads following intra-cranial administration (Wang et al., 2012). However, when such mice were infected through the foot pad, they demonstrated less viral dissemination to the brain, decreased inflammatory mediator production, reduced leukocytes recruitment to the CNS, and lower mortality, compared to that seen in wild type animals (Wang et al., 2012). As such, these seemingly contradictory findings may indicate a double-edged role for IL-22 in viral infections, where this cytokine promotes pathogen spread to the CNS, but also limits inflammatory damage within the brain once the BBB has been breached.

## The Role of IL-20 and IL-24 in the CNS Remains Unclear

While a considerable amount of evidence supports the protective immunosuppressive effects of IL-10 and IL-19 within the CNS, and at least some evidence supports a similar function for IL-22 in the brain, the role of IL-20 and IL-24 at this site remain largely unknown. Whereas IL-20 was first identified based upon a gene sequence predicted to yield a helical protein structure similar to IL-10 (Blumberg et al., 2001), the discovery of IL-24 was based upon its ability to induce apoptosis in cancer cells (Wang and Liang, 2005; Persaud et al., 2016) and this protein remains the subject of extensive research as an oncolytic therapy (Fisher et al., 2003; Sauane et al., 2003; Fisher, 2005; Buzas et al., 2011; Persaud et al., 2016; Ma et al., 2018). These studies have extended to brain cancers, including neuroblastomas and IL-24 was found to induce apoptosis in these cells when overexpressed following gene delivery using viral vectors (Bhoopathi et al., 2017).

Both IL-20 and IL-24 are expressed in myeloid cells following stimulation with TLR ligands, activated TH2 lymphocytes (Wolk et al., 2002; Rutz et al., 2014), and non-leukocytic cells such as keratinocytes (Wolk et al., 2009; Martin et al., 2017). Interestingly, the expression of IL-20 and IL-24 by keratinocytes has been reported to be induced by IL-22 suggesting an ability of IL-10 family members to function in a cooperative manner (Wolk et al., 2009; Martin et al., 2017). However, there are few reports of the expression of these cytokines within the CNS. Hypoxia has been shown to induce the expression of IL-20 mRNA and protein by glioblastoma cells (Chen and Chang, 2009), while mixed primary glia show a rapid (within 2 h) and transient expression of mRNA encoding IL-20 following challenge with bacterial LPS (Hosoi et al., 2004). Similarly, we have reported the expression of IL-20 mRNA by murine astrocytes exposed to *Neisseria meningitidis* (Cooley et al., 2014; Table 1). While IL-24 mRNA expression has also been demonstrated in murine astrocytes following alphavirus infection (Das et al., 2015) or bacterial challenge (Cooley et al., 2014), our recent observations indicate that such expression is delayed with kinetics of induction that resemble IL-10 and IL-19 (Burmeister et al., unpublished observations). Additionally, pulsed electromagnetic field treatment following cerebral ischemia has been associated with upregulated mRNA encoding IL-24 within brain tissue at 7 days post-treatment (Pena-Philippides et al., 2014).

Neither IL-20 nor IL-24 signal via either of the IL-10R subunits (Zhang et al., 2000), but unlike the other members of the IL-10 cytokine family that have been discussed thus far, IL-20 and IL-24 can both signal through two different heterodimeric receptors, IL-20 receptor types 1 and 2, which are composed of IL-20R1 and IL-20R2, and IL-22R1 and IL-20R2 subunits, respectively (Ouyang et al., 2011; Rutz et al., 2014; Figure 1). As mentioned earlier, these receptor subunits are primarily expressed by non-hematopoietic cells, and have been reported to be present in microglia, astrocytes and an astrocytic glioblastoma (Dumoutier et al., 2001; Wolk et al., 2002; Cooley et al., 2014; Horiuchi et al., 2015; Perriard et al., 2015). Following cytokine binding, these receptors initiate JAK/STAT signaling pathways in the target cell. IL-20 and IL-24 utilizes JAK1 and STAT1 or, more predominantly, STAT3, in embryonic kidney cells and colonic epithelial cells (Dumoutier et al., 2001; Parrish-Novak et al., 2002; Andoh et al., 2009), and can also initiate the phosphorylation of ERK1/2 and p38 in keratinocytes (Andoh et al., 2009; Lee et al., 2013; Hsu et al., 2015). Similarly, glioblastoma cells exposed to IL-20 demonstrate phosphorylation of STAT3, ERK and Akt (Chen and Chang, 2009). To date, however, the signaling pathways activated in glial cells by IL-24 have not been defined.

Despite the reported expression of IL-20, IL-24 and their receptors by glial cells, little is known about the function of these cytokines within the CNS. In the periphery, elevated IL-20 and IL-24 levels have been detected in the serum of patients with chronic inflammatory disorders, such as Crohn’s disease, psoriasis and rheumatoid arthritis (He and Liang, 2010; Rutz et al., 2014), and genetic polymorphisms for these cytokines have been identified as risk factors for some of these chronic inflammatory diseases (Kumari et al., 2013; Khodakheir et al., 2017). Within the CNS, inhibition of IL-20 using a neutralizing antibody has been shown to limit the inflammatory damage associated with acute ischemic brain injury (Chen and Chang, 2009), and IL-20 exposure has been demonstrated to promote the release of the potent chemoattractants MCP-1 and IL-8 by a glioblastoma cell line (Chen and Chang, 2009). In contrast, we have recently shown that IL-24 can induce the expression of SOCS3, a signaling component that inhibits the effects of IL-6, in murine astrocytes and can attenuate inflammatory mediator production by these cells following bacterial challenge (Burmeister et al., unpublished observations). Furthermore, we have determined that IL-24 can also augment the expression of IL-10 by astrocytes following activation, providing another potential means by which this cytokine could limit neuroinflammation. Clearly, much further work is needed to define the apparently opposing actions of IL-20 and IL-24 on glial immune functions.

## Concluding Remarks

Within the brain, it has become increasing apparent that glial cells contribute both to the maintenance of an immunoquiescent environment within the CNS, and to the initiation and progression of potentially damaging neuroinflammation. It is clear that both microglia and astrocytes can be a source of IL-10, and that they are responsive to the immunosuppressive actions of this cytokine (Jack et al., 2005; Bsibsi et al., 2006; Rasley et al., 2006; Park et al., 2007; Gautam et al., 2011; Gutierrez-Murgas et al., 2016). The kinetics of induction of IL-10 are consistent with a role in the resolution of glial inflammatory responses, and the association of human patient IL-10 gene polymorphisms with neuroinflammatory disorders support such a role (Martinez Doncel et al., 2002; Myhr et al., 2002; Talaat et al., 2016). Likewise, the preponderance of available evidence supports a similar function for IL-19, which demonstrates similar delayed kinetics of induction and can also limit inflammatory mediator production by glial cells (Cooley et al., 2014; Nikfarjam et al., 2014). However, the purpose of other IL-10 family members within the CNS is far less defined, with IL-22 being suggested to play a protective immunosuppressive role in some instances, and a detrimental pro-inflammatory function in others, perhaps reflecting the pleiotropic nature of these cytokines (Kebir et al., 2007; Beyeen et al., 2010; Laaksonen et al., 2014; Akil et al., 2015; Perriard et al., 2015). The limited information available for IL-24 suggests that it may act like IL-10 and IL-19, providing delayed protection during CNS inflammation, while IL-20 seems to contribute primarily to the inflammatory phase, demonstrating rapid induction kinetics (Hosoi et al., 2004).

**Figure 2:**
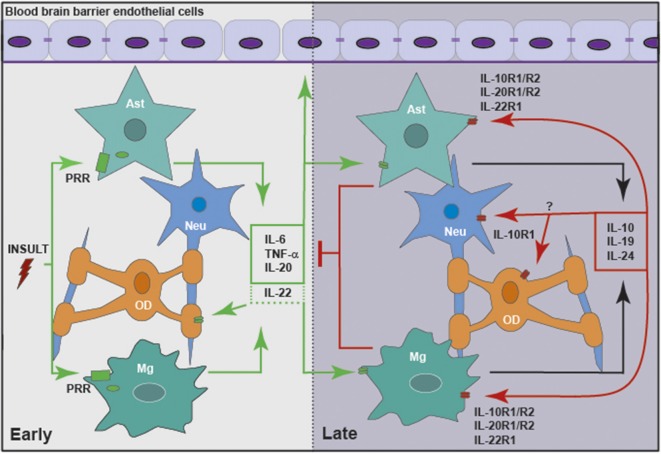
Members of the IL-10 family of cytokines are produced by glia in response to central nervous system (CNS) insult, either directly or in a delayed indirect manner, to exacerbate or limit neuroinflammation. Glial cells, including microglia (Mg) and astrocytes (Ast), respond to insult via pattern recognition receptors (PRRs), including cell surface and cytosolic receptors. Following activation, glia release pro-inflammatory cytokines, including IL-6, tumor necrosis factor-α (TNF-α), IL-20 and perhaps IL-22. These mediators act to promote the clearance of the initial insult by altering the integrity of the blood brain barrier (BBB) and recruiting leukocytes from the circulation. In addition, inflammatory mediators act in an autocrine and/or paracrine manner to promote the delayed expression of IL-10, IL-19 and IL-24 by glia. These cytokines act via their cognate receptors expressed by astrocytes and microglia, and perhaps oligodendrocytes (OD) and neurons (Neu), to curtail the inflammatory responses of these cells and/or recruited leukocytes.

However, it is evident that our current understanding of the role of IL-10 and the other members of this cytokine family within the CNS is limited at best. While it is clear that glia can be a significant source of IL-10, IL-19 and perhaps IL-20 and IL-24, and these resident CNS cells are responsive to their actions (as summarized in Figure 2), the functions of the IL-10 cytokine family in health and brain disorders have been understudied. Given the available evidence that IL-10 and its relatives are present in inflammatory diseases of peripheral organs and tissues, and that they exert a significant effect on the incidence and severity of such conditions, it is not unreasonable to assume that these cytokines are similarly important within the CNS during infection or other inflammatory brain disorders. Clearly, more research is warranted to define the actions of the IL-10 family within the CNS and their role in the regulation of neuroinflammation.

## Author Contributions

AB and IM wrote and edited this review article.

## Conflict of Interest Statement

The authors declare that the research was conducted in the absence of any commercial or financial relationships that could be construed as a potential conflict of interest.
